# Open source libraries and frameworks for mass spectrometry based proteomics: A developer's perspective^☆^

**DOI:** 10.1016/j.bbapap.2013.02.032

**Published:** 2014-01

**Authors:** Yasset Perez-Riverol, Rui Wang, Henning Hermjakob, Markus Müller, Vladimir Vesada, Juan Antonio Vizcaíno

**Affiliations:** a EMBL Outstation, European Bioinformatics Institute, Wellcome Trust Genome Campus, Hinxton, Cambridge, CB10 1SD, UK; b Department of Proteomics, Center for Genetic Engineering and Biotechnology, Ciudad de la Habana, Cuba; c Proteome Informatics Group, Swiss Institute of Bioinformatics, CMU - 1, rue Michel Servet CH-1211 Geneva, Switzerland

**Keywords:** AMT, Accurate Mass Tag, ATAQS, Automated and Targeted Analysis with Quantitative SRM, CV, Controlled Vocabulary, DAO, Data Access Object, EBI, European Bioinformatics Institute, emPAI, exponentially modified Protein Abundance Index, FDR, False Discovery Rate, (HUPO)-PSI, (Human Proteome Organization) — Proteomics Standards Initiative, GUI, Graphical User Interface, ICAT, Isotope-Coded Affinity Tags, ICPL, Isotope-Coded Protein Label, IPTL, Isobaric Peptide Termini Labeling, ISB, Institute for Systems Biology, iTRAQ, Isobaric Tag for Relative and Absolute Quantitation, JPL, Java Proteomic Library, LC-MS, Liquid Chromatography–Mass Spectrometry, LIMS, Laboratory Information Management System, MGF, Mascot Generic Format, MIAPE, Minimum Information About a Proteomics Experiment, MS, Mass Spectrometry, SILAC, Stable Isotope Labeling by Amino acids in Cell culture, PASSEL, PeptideAtlas SRM Experiment Library, PRIDE, PRoteomics IDEntifications (database), PSM, Peptide Spectrum Match, PTM, Post-Translational Modifications, RT, Retention Time, SRM, Selected Reaction Monitoring, TMT, Tandem Mass Tag, TOPP, The OpenMS Proteomics Pipeline, TPP, Trans-Proteomic Pipeline, Proteomics, Databases, Bioinformatics, Software libraries, Application programming interface, Open source software

## Abstract

Data processing, management and visualization are central and critical components of a state of the art high-throughput mass spectrometry (MS)-based proteomics experiment, and are often some of the most time-consuming steps, especially for labs without much bioinformatics support. The growing interest in the field of proteomics has triggered an increase in the development of new software libraries, including freely available and open-source software. From database search analysis to post-processing of the identification results, even though the objectives of these libraries and packages can vary significantly, they usually share a number of features. Common use cases include the handling of protein and peptide sequences, the parsing of results from various proteomics search engines output files, and the visualization of MS-related information (including mass spectra and chromatograms). In this review, we provide an overview of the existing software libraries, open-source frameworks and also, we give information on some of the freely available applications which make use of them. This article is part of a Special Issue entitled: Computational Proteomics in the Post-Identification Era. Guest Editors: Martin Eisenacher and Christian Stephan.

## Highlights

• A review of existing open-source software for computational proteomics.
• Available software for each step in a typical MS experiment is described.
• OpenMS, TPP, compomics, ProteoWizard, JPL, PRIDE toolsuite are covered in detail.
• Different programming languages are considered (Java, Perl, C++ or Python).

## 1 Introduction

Mass spectrometry (MS)-based proteomics has become an increasingly prominent field in the last decade, allowing the identification, quantification and characterization of peptides and proteins in biological samples [1,2]. Developments of technology and methodology in the field have been rapid over the last years and are providing improved and novel strategies for the global understanding of cellular function. Different strategies for peptide and protein identification are followed by the different experimental approaches available. In the bottom-up approaches, complex protein mixtures are enzymatically digested into potentially very complex peptide mixtures, which are then fractionated by multidimensional chromatography steps before they are subjected to tandem MS [3]. Currently, this is the most used strategy. In the top-down approaches [4], intact proteins are measured and different isoforms can be isolated before the MS identification and characterization are performed. This is especially useful to unravel complex patterns of splice variations, or post-translational modifications (PTMs) [4]. Finally, the targeted proteomics approaches [5] differ fundamentally from the previous two approaches, since the mass spectrometer is here programmed to detect and analyze only pre-selected proteins. The most popular targeted approach is called SRM (Selected Reaction Monitoring). In addition, quantification techniques can measure the differences in protein expression between different physiological states of a biological system. Nowadays, MS-based techniques comprise some of the most used quantitative approaches [6].

The advances in the MS proteomics methods are closely related to the parallel developments that have happened in bioinformatics. Several computational methods can now be used to identify peptides and proteins. The most popular ones are based on the use of search engines [7] and protein sequence databases, but there are other approaches such as *de novo* sequencing (especially used when the genome of the studied organism is not well known) [8,9] and the spectral library searches [10,11]. As a result, there are several well established software applications like Mascot [12], X!Tandem [13], Sequest [14], MyriMatch [15], SpectraST [11], OMSSA [16], and Andromeda [17], among others.

However, there is an increasing demand for high-performance bioinformatics solutions that can help to address the various data processing and data interpretation challenges in the field [18–20]. And while these tools can vary substantially, a basic set of features can be shared between many of them. Common MS data processing tasks comprise theoretical analysis of proteomes, processing of raw spectra, file format conversions, generation of identification statistics, and the storage/visualization of raw data, identification and quantitation results. As a consequence, the number of available open-source software libraries and frameworks has increased significantly in recent years. These platforms provide common software infrastructures, features and algorithms that can help in the development of new applications and tools. Previous reviews addressed the advances in the field of software tools and bioinformatics applications for proteomics MS experiments [18,19,21–23], but open-source frameworks and libraries were not evaluated in detail. There are now a wide variety of software solutions covering all aspects of LC–MS/MS data analysis, which are developed and maintained by an active community of bioinformaticians and software developers [23]. In this review we focus mostly on open-source frameworks, software libraries and downloadable tools, so most of the existing online resources have not been included. The R programming language will not be considered here either since it is covered in another manuscript in this special issue. We will follow the steps of a typical tandem MS proteomics workflow to describe the available software suitable for each of them. In addition, we will mention some tools that are specific for targeted proteomics approaches (SRM).

## 2 Tandem MS proteomics workflow and open-source software

**Fig. 1:**
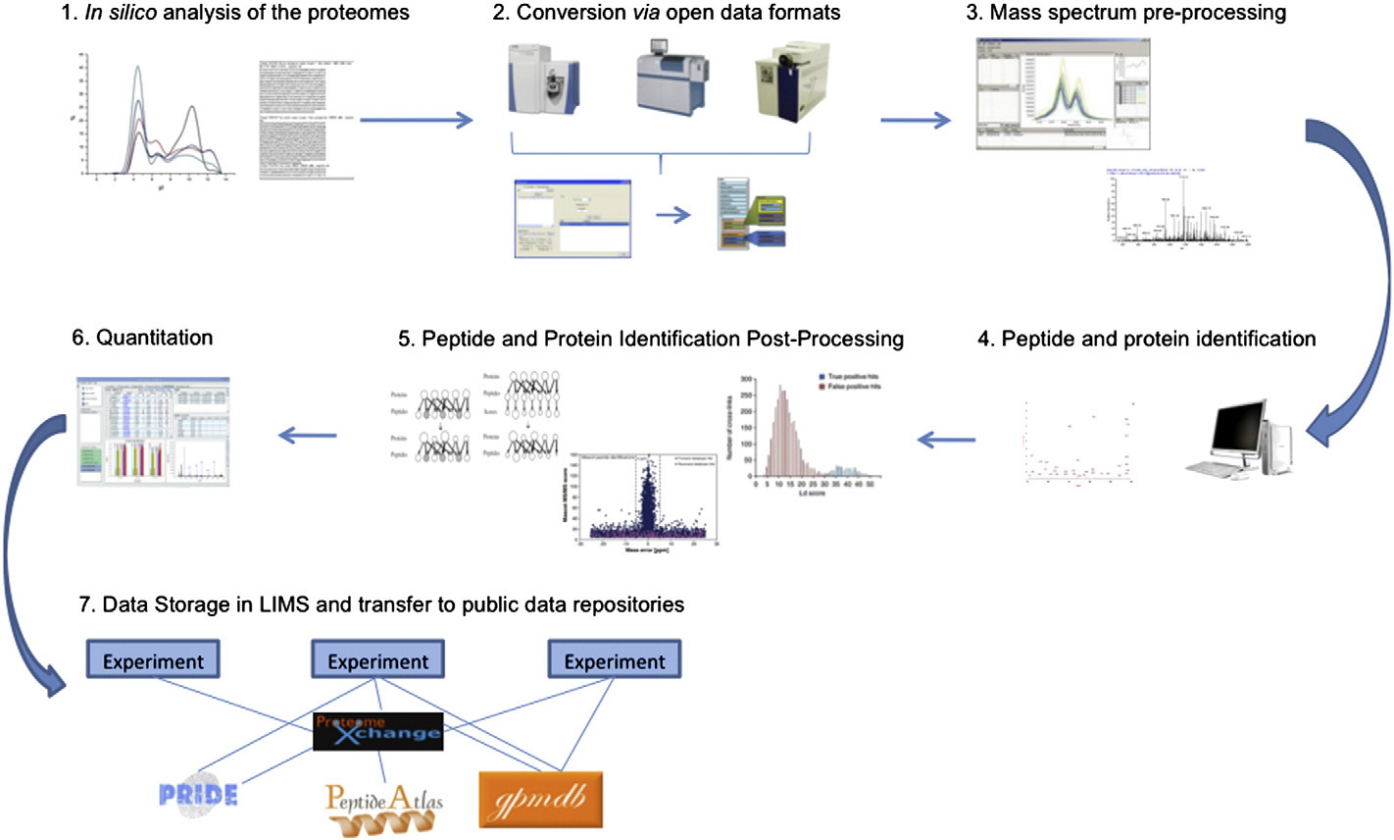
Schema of the possible computational processing steps of a proteomics data set.

A typical tandem MS proteomics experiment starts with the isolation of proteins from the sample or samples of interest [24–26]. Different approaches are used to reduce the complexity of samples such as the electrophoresis-based [27–29] and chromatography-based workflows [30–32]. As the peptides are injected into the mass spectrometer, the instrument first acquires a precursor ion scan, wherein each intact peptide ion produces a peak in the mass spectrum. A mass spectrum of the fragment ions, known as a tandem mass (MS/MS) spectrum, is then obtained for each selected precursor. A typical analysis of experimental data coming from a MS/MS study will involve most if not all of the following seven steps (Fig. 1): 1) *In silico* analysis of proteome/sequence databases, 2) conversion of raw data to open data formats, 3) mass spectrum pre-processing, 4) peptide and protein identification, 5) peptide and protein identification post-processing, 6) quantification analysis, and 7) data storage in a LIMS and transfer to public data repositories (Fig. 1).

Some of the most popular and most extensively used open-source frameworks are OpenMS [33], the Trans-Proteomic Pipeline (TPP) [34], the Computational Omics (Compomics) suite [35–39], the PRoteomics IDEntifications (PRIDE) database toolsuite [40,41], ProteoWizard [42] and the Java Proteomic Library (JPL) [43,44]. Other well-known libraries/frameworks with a more specialized scope include *InsilicoSpectro* [45], *multiplierz* [46], *mMass* [47], *mzMine* [48], *msInspect* [49], *MSQuant* and *MASPECTRAS* [50]. The aims and functionalities of each framework and library are explored in detail in the following sections.

### 2.1 Highlights of the main open-source libraries and frameworks

#### 2.1.1 OpenMS

OpenMS is a software framework for enabling rapid application development in MS. It has been designed to be portable and robust while offering rich functionalities, ranging from the availability of basic data structures to sophisticated algorithms for data analysis. OpenMS (http://open-ms.sourceforge.net/) is mainly developed in C++ and makes use of several external libraries such as: (i) Qt (http://qt.nokia.com/products/), which provides visualization and database support; (ii) Xerces (http://xerces.apache.org/xerces-c/) for XML file parsing; (iii) libSVM (http://www.csie.ntu.edu.tw/~cjlin/libsvm), for machine learning algorithms; and (iv) the GNU Scientific Library (GSL, http://www.gnu.org/software/gsl/), used for mathematical and statistical analysis. The framework architecture consists of several layers, a core application programming interface (API), which captures the MS data and complementary metadata, and a higher-level functionality API that contains database I/O, file I/O and other analysis algorithms.

The framework contains a complete set of examples to extend and use the libraries. In particular, the package for signal processing provides several filters to reduce the chemical and random noise, as well as the baseline trends in the MS measurements. In addition, the quantitation package allows the analysis of different samples using SILAC, iTRAQ and label-free algorithms [33]. Finally, the TOPP (The OpenMS Proteomics Pipeline) and TOPPView tutorials describe in detail the OpenMS tools and the user interface. Also, they provide a complete list and the corresponding command line interfaces of all the TOPP tools contained in each release.

#### 2.1.2 Trans-Proteomic Pipeline

The Trans-Proteomic Pipeline (TPP, http://sourceforge.net/projects/sashimi/) [34] contains several very popular tools in the field. Developed at the Institute for Systems Biology (ISB, Seattle, USA), the framework comprises a set of components, libraries and tools. They encompass most of the steps involved in a proteomics data analysis workflow in a single, integrated software system, including mass spectrometer output file conversion, protein identification statistical validation, quantification by stable isotope ratios, and support for SRM. To summarize the pipeline, raw mass spectrometer output files are first converted to open XML standard formats. These files are run through one or more search engines such as X!Tandem, Mascot, Sequest, or SpectraST.

PeptideProphet [51], iProphet [52], and ProteinProphet [53] can then be used to validate the search engine results and to model correct vs. incorrect peptide-spectrum matches (PSMs) and the protein inference. The quantification analysis tools XPRESS [54] or SuperHim [55] may then be applied with data that derive from labeled or label-free quantitation approaches. In addition, mProphet and mQuest can automate the analysis of SRM data, and provide probabilistic scoring of targeted peptide identifications and derived quantification [56].

The TPP components have been developed using different programming languages such as C++, Perl and Java. This fact complicates the integration with other pieces of code and the development of new applications using the TPP framework.

#### 2.1.3 Computational Omics (Compomics)

The Compomics framework is an independent platform and pure Java package with a common core API for all the libraries and tools [35]. The platform source code, documentation and tools, along with a complete set of examples are freely available at http://compomics-utilities.googlecode.com. The framework contains a set of parsers for popular search engines output files (Mascot, X!Tandem, OMSSA and Proteome Discoverer (Thermo Scientific)). It also includes a collection of user-friendly tools, including among others: (i) ms_lims [36] and DBToolkit [39] for storing and performing different *in silico* analysis of proteomics data; (ii) Peptizer [38] for manual validation of MS/MS search results; (iii) Rover [57], for visualizing and validating quantitative proteomics data; (iv) FragmentationAnalyzer [37] for analyzing MS/MS fragmentation data; (v) the new PeptideShaker (http://peptide-shaker.googlecode.com), for comprehensive MS data combined analysis of results from multiple search engines (Mascot, OMSSA and X!Tandem); and (vi) SearchGUI [58], which provides a unified GUI (Graphical User Interface) for MS identification using multiple search engines (OMSSA and X!Tandem).

#### 2.1.4 ProteoWizard

The ProteoWizard framework provides a modular and extensible set of open source, cross-platform tools and libraries [42]. This platform enables rapid tool creation and unifies data file access to perform standard proteomics and LC–MS analysis computations. Developed in C++, it is cross-compiled and freely available at http://proteowizard.sourceforge.net/. ProteoWizard provides multiple independent libraries, which are grouped together at different levels. The framework includes different tools for data conversion and a core API for parsing different data formats. In addition to the open mzML [59], mzXML [60], mzIdentML [61], and mzData XML formats, a variety of proprietary formats can also be handled. As a result, several frameworks/applications such as TPP and Skyline [62,63] extend and use the ProteoWizard core APIs and tools. The C++ source code is designed and optimized for high performance and high throughput analysis, and allows researchers to implement novel algorithms or to complete other *ad hoc* tasks.

#### 2.1.5 Java Proteomic Library (JPL)

The Java Proteomic Library (JPL, http://javaprotlib.sourceforge.net/), developed in Java by the Swiss Bioinformatics Group (Geneva, Switzerland) provides a strong chemical-based representation of MS proteomics data. It is composed of several modules and APIs for manipulating peptide or protein sequences, PTMs and mass spectra. It also provides methods for *in silico* protein digestion and peptide fragmentation, which takes into account various ion types and modifications. Many classes dealing with spectrum processing and filtering, and/or spectrum matching and clustering, are also provided. The availability of core classes that represent modifications, peak annotations and chemical entities in a proteomics context, makes JPL the ideal framework to compute physicochemical properties, such as isoelectric point (*pI*), retention time (RT) and gravy index. In addition, it also contains several standalone tools for performing protein sequence digestion, creating spectrum and sequence decoy databases [43], and performing open modification spectrum library searches (*QuickMod/Liberator*) [44]. JPL is currently being refactored in order to increase its performance, improve structure of classes and the amount of ‘Unit’ tests available. A new version will be officially released once all this work has been finished. The JPL is well-documented and contains different examples about how to use some of its classes.

#### 2.1.6 PRIDE toolsuite

The PRIDE database was developed at the European Bioinformatics Institute (EBI), as a repository to store the experimental results from bottom-up MS-based proteomics experiments [40]. The PRIDE toolsuite (http://pride-toolsuite.googlecode.com) constitutes a set of pure Java libraries, tools and packages designed to handle MS proteomics experiments from a vast range of approaches, instruments and analysis platforms. The framework contains a set of components such as: (i) the *mzGraph Browser* library, for visualizing MS spectra, chromatograms and MS/MS spectrum annotation; (ii) the *QualityChart* library provides a number of charts for performing a quick quality assessment of the MS experiments; (iii) several APIs for parsing standard data proteomics formats such as mzML, mzIdentML, mzTab and PRIDE XML (the PRIDE internal data format); (iv) the *XXIndex* library enables the fast indexing of large XML files; (v) the *PRIDE Utilities* library contains classes with some functionality shared by many of the PRIDE related tools; and (vi) the *PRIDE core* library (http://ebi-pride.googlecode.com), for general data management.

The PRIDE Converter 2 [64] and the PRIDE Inspector [41] are currently the most popular tools of the framework, and both of them offer a user-friendly GUI. PRIDE Converter 2, recently released, is a new submission tool for converting a large variety of popular MS proteomics formats into PRIDE XML, by guiding the user through a wizard-like process. A command line mode (CLI) mode is also available for converting multiple files at once in batch mode. Its predecessor, the original PRIDE Converter tool [64], is currently being phased out, since the new software has been made available. Finally, PRIDE Inspector is a tool that allows the user to efficiently browse, visualize, and perform an initial assessment of MS proteomics data in the PRIDE XML and mzML [59] formats, and also allows direct access to a PRIDE MySQL public database instance. Support for the formats mzIdentML and mzTab is in progress. Finally, the most recent addition to the PRIDE-toolsuite is the *PRIDE spectra clustering* API (http://pride-spectra-clustering.googlecode.com).

### 2.2 *In silico* analysis of the proteome and sequence databases

Proteomics experiments targeting specific proteins need to carefully choose the approaches used in order to maximize the possibility that the proteins of interest are present and can be identified [23]. For instance, to perform *in silico* studies of proteomes and sequence databases can enable the optimization of the experimental settings [65]. Also, the study of the identified proteins is crucial to predict the protein and peptide properties needed for performing targeted proteomics experiments such as SRM.

The features needed for analyzing protein sequence databases are fortunately well represented in the existing software libraries. For example proteolytic digestion, property estimation (*pI*, retention time, hydrophobicity, etc.) and amino acid distribution are some of these common features. Certain properties can then be used to design a targeted proteomics workflow to detect proteins, which are often missed in the typical workflows.

A significant number of theoretical analyses about the relationships between the *pI* and different protein properties such as length, taxonomy or hydrophobicity have been published [65–68]. Also, different *in silico* analyses of the proteome for performing accurate mass and time (AMT) tag [69] approaches, the decoy method studies [70], and the analysis of different isolation methods combined with accurate mass [71], are good examples of theoretical proteomics analysis as well. Table 1 shows a list of software libraries that can be used for the *in silico* analysis of proteins.

#### 2.2.1 OpenMS

The OpenMS framework offers functionalities for analyzing both the protein sequence databases and the identification results. It provides different functions for predicting sequence properties (retention time, *pI*, mass, etc.) and reading protein databases from FASTA files.

#### 2.2.2 Compomics

The DBToolkit from the Compomics framework (http://dbtoolkit.googlecode.com) provides a GUI to build sequence databases, after performing different processing steps such as protease digestion, decoy and sequence pattern filtering. In addition, the *compomics-utilities* library can be used programmatically to read and parse FASTA files, perform *in silico* digestion, and predict sequence properties.

#### 2.2.3 Java Proteomic Library (JPL)

The JPL provides different functions for predicting the *pI* of peptides and proteins with several experimental settings. It also provides: (i) the *MassCalc* tool to compute masses for proteins or molecules; (ii) *ProteinDigester* to perform digestion of proteins (Supplementary Information), compute the *pI* and molecular weight of all the digested peptides; and (iii) *Dig2Mz* to perform protein digestion and compute the *m/z* values of all digested peptides that passed the charge filters.

#### 2.2.4 Other tools, packages and open-source frameworks

*InsilicoSpectro* [45] was developed in Perl and offers different sets of functionalities, for instance protein digestion, sequence database file readers, property estimation (*pI*, retention time, mass) and MS fragmentation prediction. Different groups have used extensively this library [68,71–73] due the availability of several database file readers, and the possibility to predict different physicochemical properties in heterogeneous experimental settings. *Database on Demand* (http://www.ebi.ac.uk/pride/dod/) [74] is a Java web application that can be used to design customized search databases that provide detailed control over the search space.

Python is not an extensively used programming language in computational proteomics, but in recent years is gaining popularity. Then, *Multiplierz* [46] and *Pyteomics* (http://pypi.python.org/pypi/pyteomics/) are frameworks to support proteomics data analytic tasks in this language. Access to the available functionality is provided *via* high-level Python scripts. Already mentioned features such as the availability of sequence database file readers and the prediction of different physicochemical properties (*pI*, retention time, mass) are present in both libraries [46,75,76]. *Pyteomics* is fully integrated and currently indexed in the Python Package Repository (PyPI).

### 2.3 MS file parsers and conversion

#### 2.3.1 Mass spectrometry file formats

The primary data content produced in the context of a MS-based proteomics experiment are the mass spectra. Each mass spectrometer vendor uses different proprietary file formats to store the spectra produced [31,77]. The structure of the data varies depending on the instrument and the experiment type, and the files typically consist of MS1 spectra interleaved with multiple MS/MS spectra. The “aging” issue (as time passes, support for certain formats tends to disappear) and the “binary” character of the files (proprietary software dependency) are two of the main limitations of these file formats. This led to the creation of different XML-based open standard formats [78], since it is impractical for software tools developed for general use to support all these different formats. Since then, the development of such formats has enabled a significant increase in MS data sharing [40] and validation [79].

**Fig. 2:**
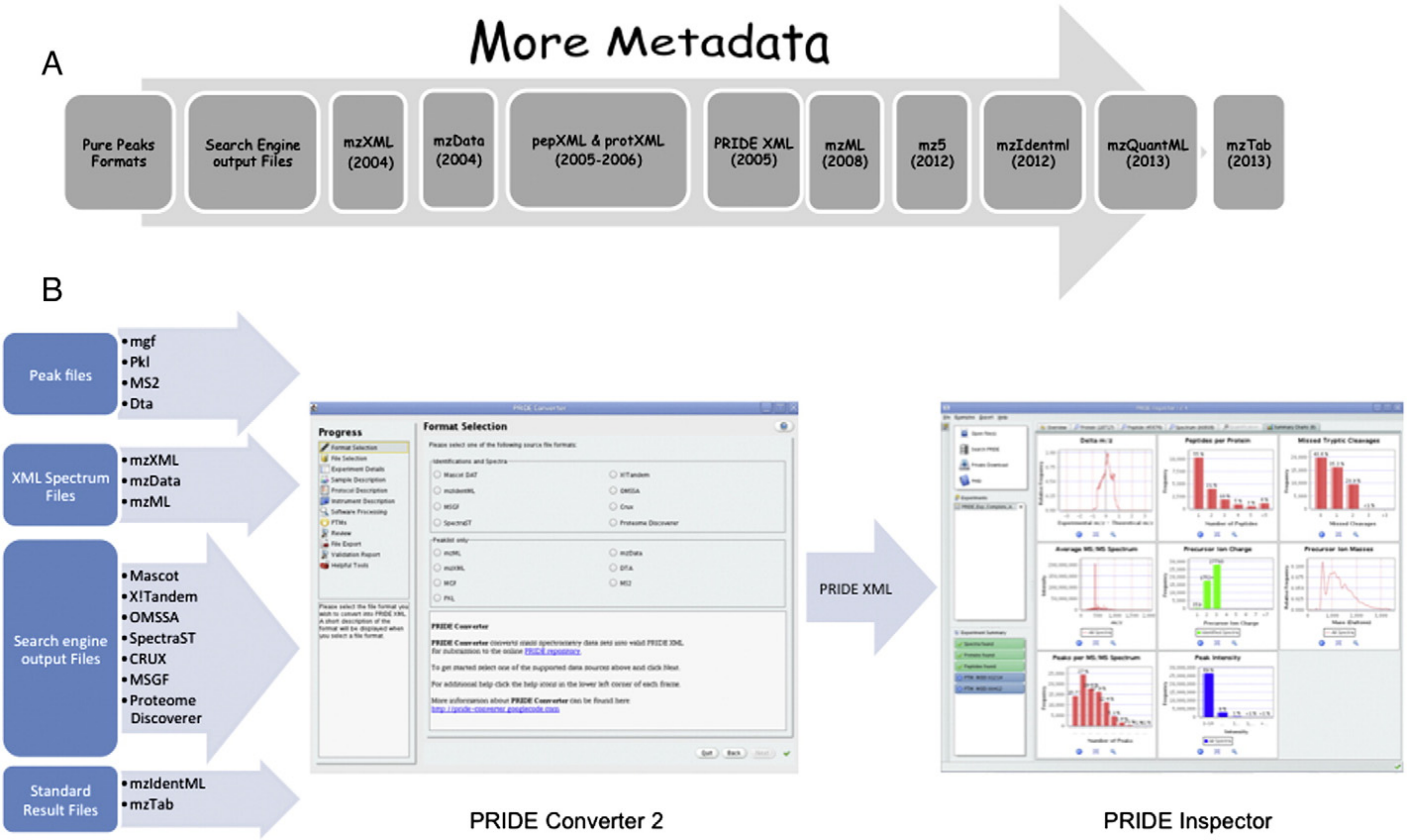
(A) Evolution of Mass Spectrometry file formats. (B) Schema of the PRIDE toolsuite tools PRIDE Converter 2 and PRIDE Inspector.

Fig. 2A shows the evolution of different MS file formats in recent years. For instance, mzXML [60], developed by ISB, was one of the first initiatives quickly adopted by the community. In recent years, the HUPO Proteomics Standards Initiative (PSI) has developed a set of important community XML file formats such as mzML (for MS data) [59], mzIdentML (for peptide/protein identifications) [61], and gelML (for gel data) [80], for proteomics data storing, representation and visualization. Recently, TraML [81] has been developed as a standard format for encoding transition lists and associated metadata. Quantitative data can be encoded in the nascent formats mzQuantML (XML-based, http://mzquantml.googlecode.com) and a text-based tab-delimited file called mzTab (http://mztab.googlecode.com).

#### 2.3.2 ProteoWizard

The main tools included in ProteoWizard are: (i) *msConvert,* for data conversion from vendor proprietary formats to mzML and mzXML; (ii) *msDiff,* to compare two data files; and (iii) *msAccess*, providing command line access to MS data files (such as mzML, Supplementary Information).

The *msConvert* tool is a very popular application that can convert MS data in several proprietary formats such as .WIFF (ABI/Sciex), .BAF (Bruker), .RAW (ThermoFisher Scientific), .D (Agilent) and others, into a mzML, mzXML, mz5 [82] (a reimplementation of mzML, based on the efficient, industrial storage backend HDF5, http://www.hdfgroup.org/HDF5), and the text based formats MGF (Mascot Generic Format) and ms2. Annotation in the mzML files is encoded using ‘CVParam’ elements, which refer to the terms present in a given controlled vocabulary (CV) or ontology. ProteoWizard parses the CV/ontology file at compile time and generates C++ code, which allows convenient, typesafe handling of the CV terms.

#### 2.3.3 PRIDE toolsuite

PRIDE Converter 2 supports the conversion from different popular data formats into PRIDE XML. The PRIDE Converter 2 framework, as part of PRIDE toolsuite, consists of four different components: *PRIDE Converter 2*, *PRIDE mzTab Generator* (to generate mzTab files), *PRIDE XML Merger* (to merge results from different PRIDE XML files into a single file) and *PRIDE XML Filter* (to filter out some of the data present in the files). At present, the PRIDE Converter 2 supports several file formats: Mascot, X!Tandem, OMSSA, mzIdentML, SpectraST [83], CRUX [84], MSGF [85], Proteome Discoverer, mzML, dta, MGF, mzData, mzXML, and pkl. New file formats can be supported simply by implementing the Java *DAO* (Data Access Object) interface. Fig. 2B shows how the PRIDE toolsuite tools (PRIDE Converter 2 and PRIDE Inspector) can be combined.

As mentioned before, the PRIDE toolsuite also contains several Java APIs for read/write several standard formats, some of which are used in PRIDE Converter 2 and PRIDE Inspector, but also in other external software: *jmzML* [86] for mzML, *jmzIdentML* [87] for mzIdentML, and the new *jmzTab* (http://mztab.googlecode.com) to read and write mzTab files. Both *jmzML* and *jmzIdentML* use the *XXindex* library (http://pride-toolsuite.googlecode.com), an indexing system for large XML files retrieving, allowing a random access to the data.

Recently, the *jmzReader* [88] library was developed providing a common programming interface for different XML based and/or peak list formats such as: MGF, ms2, dta, mzData, mzXML, pkl, and mzML. This Java library provides functions to randomly access spectra within the files without the need to load the whole file into memory, and allows easy integration with mzIdentML.

#### 2.3.4 Compomics

The Compomics framework provides different Java-based parsers for well-known search engines: *MascotDatfile* [89], *OMSSA Parser* [90], *XTandem Parser* [91], and *Thermo-MSF-Parser* [92], for Proteome Discoverer. It also provides the *jTraML* [93] library for the TraML standard file format. Also, *PeptideShaker* supports the creation of a well-annotated PRIDE XML file from the combined search result files from Mascot, OMSSA and X!Tandem.

#### 2.3.5 Other packages and open-source frameworks

Table 2 shows different libraries that can be used to read and write MS files formats (both peak list and peptide/protein identification files). The JRAP (http://sashimi.sourceforge.net/software_glossolalia.html#JRAP) library was written in Java at the ISB, and has been historically the most extensively used library to handle mzXML files.

Other well-focused libraries are *MGFp* [94], *pyMzML* [95], which enable read/write operations in MGF and mzML files, respectively. In addition, the OpenMS and TPP frameworks support mzML, mzXML, MGF and output files from the search engines Mascot, Sequest and X!Tandem.

### 2.4 Mass spectrum pre-processing

Mass spectrum preprocessing algorithms can increase the number of identified peptides and improve the reliability of the peptide identifications. Five types of pre-processing methods are widely used: spectrum normalization, spectrum clustering, precursor charge determination, spectrum de-noising, and spectrum quality assessment [96]. It is worth noticing that these algorithms are also applicable to MS-based metabolomics approaches, which are also being increasingly applied to characterize biological systems.

The basic aim of the data pre-processing steps is to transform the raw MS data files into a file format that facilitates an easy access to the characteristics of each observed ion. These characteristics include *m/z* values, retention time and ion intensity measurements present in the original raw data files. In addition to these basic features, data preprocessing can extract additional information like the isotope distribution of the ions. Table 3 shows some of the most useful libraries for MS proteomics and metabolomics data preprocessing.

#### 2.4.1 OpenMS

OpenMS provides several filters for noise reduction (also called smoothing filters). Among them, a Gaussian filter, the Savitzky–Golay filter and the baseline correction (Supplementary Information). Combining the possibility to read several MS file formats and several preprocessing peak algorithms make the OpenMS framework a versatile and complete environment for MS preprocessing.

#### 2.4.2 Java Proteomic Library (JPL)

JPL implements many MS processing methods, ranging from peak intensity transformations to noise reduction filters. The library also supports peak annotations and different file formats such as mzML, mzXML, and MGF.

#### 2.4.3 Other packages and open-source frameworks

*mMass* [47], is a cross-platform software library that can be used for the precise analysis of individual mass spectra. Even when the library was not designed for high-throughput MS analysis, its Python API offers the foundation to develop new tools for MS preprocessing. The software library covers a wide range of processing tasks such as smoothing, baseline correction, peak picking, deisotoping, charge determination, and recalibration. Especially developed for analyzing MS experiments of lipids, a leading feature is the implementation of the lipid database obtained from LIPID MAPS [97].

*MZmine2* [48] and *msInspect* [49] are Java libraries mainly implemented for MS preprocessing purposes. They implement solutions for several stages of MS processing such as spectral filtering, peak detection, chromatographic alignment and normalization. *Mzmine2* also provides several data mining algorithms (principal component analysis, clustering and log-ratio analysis) to reduce the dimensionality of the data. Also, the *msInspect* platform includes utilities for calculating various summary statistics in Java, and for performing linear regression using an interface with the R statistical language. Finally, the ‘Modular Application Toolkit for Chromatography Mass-Spectrometry’ (*maltcms*) library [98], written in Java, provides reusable, efficient data structures, and the capability to abstract information from the data formats mzXML, mzData and mzML, giving a consistent access to data features like mass spectra, chromatograms and metadata.

### 2.5 Peptide and protein identification post-processing

Several post-processing strategies have been developed to refine the initial peptide/protein identification list, often relying on orthogonal information not used by the identification software. These software libraries/applications, including the well-known PeptideProphet/ProteinProphet [51,53] (part of the TPP), Percolator [99,100], and Peptizer [38], essentially attempt to emphasize the score differences between correct and incorrect matches by examining various properties of the PSM assignments. This step is necessary to increase the confidence on the final reported results.

#### 2.5.1 OpenMS

OpenMS can improve the identification accuracy for several search engines and consensus identifications can be calculated from the initial results. The identifications can also be validated using retention time prediction algorithms and the *IDFilter* package can be used to filter out false positive identifications.

#### 2.5.2 TPP

TPP provides PeptideProphet, iProphet and ProteinProphet: three tools for peptide and protein identifications validation. The C++ source code of the applications is also available. These tools use the expectation maximization algorithm to separate correct from incorrect identifications based on a limited set of rules (one of the dominant properties, for instance, is the tryptic correctness of the peptide termini). The integration of the tools in TPP increases the number of correctly identified peptides at a constant false discovery rate (FDR). ProteinProphet is used to address the protein inference problem by applying a mixture model based on the number of distinct peptides per protein (sibling peptides) to boost the probabilities of peptides with multiple siblings, while penalizing peptides without siblings. Each protein is then assigned a probability of being present in the sample, based on the number of sibling peptides. ProteinProphet creates a list of proteins that can explain all the peptide observations. Recently, iProphet was added in combination with PeptideProphet to TPP. It combines the evidence from multiple identifications of the same peptide sequences across different spectra, experiments, precursor ion charge states, and modified states. It also allows accurate and effective integration of the results from multiple database search engines applied to the same data.

#### 2.5.3 PRIDE toolsuite

Very recently, the *PRIDE spectra clustering* API (http://pride-spectra-clustering.googlecode.com) has been added to PRIDE toolsuite [101]. The clustering algorithm is a modification of MS-cluster [102] and has been used to cluster all identified spectra in PRIDE. The idea behind is to give quality assessments of the PSMs stored in PRIDE and the generation of spectral libraries from highly heterogeneous data (http://www.ebi.ac.uk/pride/cluster/libraries).

#### 2.5.4 Compomics

Peptizer [38] (http://peptizer.googlecode.com) is an expert system that relies on user defined and configured expert rules to pick out suspect identifications which can then be manually evaluated or automatically rejected. Expert manual validation of the identifications is a more commonplace strategy for quality control in those cases where a subset of all peptide identifications obtained is of relevance to the biological system (Supplementary Information). *PeptideShaker* merges the identifications from multiple search engines (Mascot, OMSSA and X!Tandem) into a single result, and validates the protein, peptides and PSMs at 1% FDR. This approach dramatically increase the number of validated identifications compared to using a single search engine or using a consensus hit. The user can also analyze and alter the statistics in various ways to tailor the results. All the functionality is accessed *via* a simple and user-friendly GUI.

#### 2.5.5 Java Proteomic Library (JPL)

The QuickMod [44] tool as part of the JPL estimates the occurrence of PTMs after careful analysis of an extensive list of spectral similarity measures. The authors have showed how spectra from peptides carrying distinct modification types have different scoring characteristics, and evaluated the final scoring scheme per modification type. This tool, based on spectral clustering techniques, can be used after or in combination with database search approaches. The performance of the QuickMod algorithm was compared with the InsPecT-PTMFinder [103] software and the results showed a significant improvement in the number of identified PTMs with QuickMod [44].

#### 2.5.6 Other packages and open-source frameworks

The determination of the peptide false discovery rate using decoy databases is the most common approach used to identify false positive assignments. An alternative approach is to use machine learning methods [51,52,104] to re-rank the PSMs, based on peptide properties and search engine scores. The Percolator [104] approach first developed for the search engine Sequest, trains a machine learning algorithm called support vector machine (SVM) to discriminate between positive and negative PSMs. The algorithm, developed in C++, is open source (http://per-colator.com) and several examples are provided with the tool.

Mascot Percolator [100] is a Java library and tool designed for the validation of Mascot identified peptide/protein identifications. The algorithm, as the original percolator algorithm [104], is based on a semi-supervised SVM approach, and is able to discriminate between correct and incorrect identifications by assigning weights to a number of features such as: Mascot score, precursor mass error, fragment mass error, number of variable modifications used in the search, etc. The self-boosted Percolator [105] Java package (http://self-boosted-percolator.googlecode.com) is an extension of the original algorithm. The main improvement is the application of a cascade learning procedure to boost the algorithm to an optimal and stable state. Self-boosted Percolator is specifically designed for X!Tandem results coming from the TPP.

Using the *msInspect* framework, Damon and coworkers [106] presented a complete set of new algorithms and a software implementation for assigning confidence to peptide sequence assignments obtained through classic accurate mass and retention time (AMT) matching techniques. The algorithms increased the number of peptides and proteins identified among related proteomics experiments that use high-resolution MS instrumentation.

Finally, The FDRAnalysis [107] (http://web-based-multiplesearch.googlecode.com) is a Java library which enables the upload of peptide identification results from target/decoy searches carried out by three different search engines: Mascot, OMSSA and X!Tandem. Importantly, FDRAnalysis can import native format search results, and supports mzIdentML.

Several other algorithms and libraries have been developed to solve the protein inference problem [108]. IDPicker [109] (http://fenchurch.mc.vanderbilt.edu/) is an open source protein assembly tool that derives a minimum protein list from peptide identifications filtered to a specified FDR and increase confident peptide identifications combining multiple search engine scores. The latest version is more robust against false positive proteins, especially in searches using multispecies databases, by requiring additional novel peptides in the parsimony process. PeptideClassifier [110] is a novel, deterministic peptide classification and protein inference scheme that takes into account the gene model–protein sequence–protein identifier relationships. Each peptide sequence is classified according to its information content with respect to protein sequences and gene models. The corresponding algorithm and open source library (http://www.mop.unizh.ch/software.html) were developed in Java. PeptideClassifier can classify shotgun proteomics data from any organism presented on popular databases such as FlyBase [111], Ensembl [112] and RefSeq [113].

Finally, Barista [84,114] is a protein identification algorithm that combines two different steps (PSM verification and protein inference) into a single learning algorithm. The algorithm produces as output three ranked lists of proteins, peptides and PSMs, based on how likely the proteins and peptides are to be present in the sample and how likely the PSMs are to be correct. The algorithm was implemented in C++ and the source code and binaries are available at http://noble.gs.washington.edu/proj/crux/barista.html.

### 2.6 Quantification

#### 2.6.1 Quantification methods

**Fig. 3:**
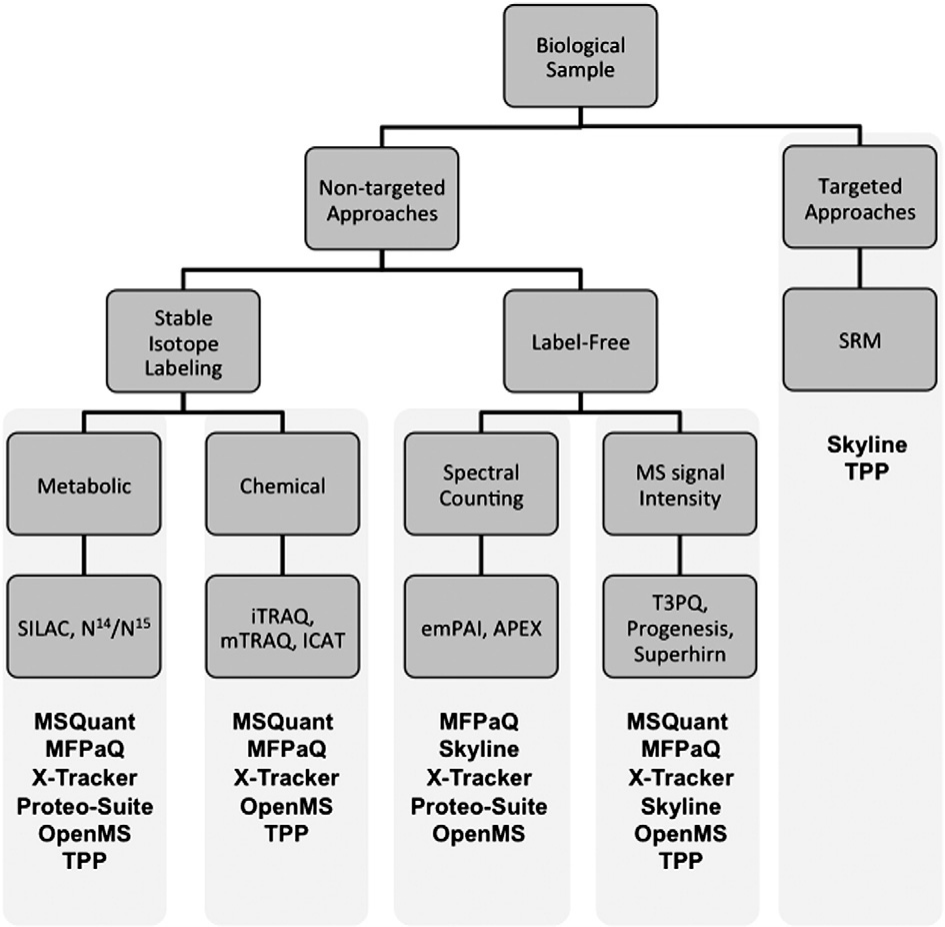
Classification of MS-based quantification methods including the open-source packages available for each of them.

Traditional MS-based quantification methods employ differential stable isotope labeling to create a specific mass tag that can be recognized by a mass spectrometer, which provides the basis for quantification [115,116]. In these methods mass spectrometers recognize the mass difference between the labeled and unlabeled forms of a peptide, and quantification is achieved by comparing their respective signal intensities. They can be introduced as an internal standard into aminoacids either (i) metabolically, or (ii) chemically (Fig. 3).

In contrast, label-free methods aim to compare two or more experiments by (i) comparing the mass spectrometric signal intensity for the identified peptides, or (ii) using the number of acquired spectra matching to a peptide/protein (spectral counting).

It is not trivial to choose an appropriate software package for the analysis of quantification data generated by a specific instrument [117]. There are three main issues: (i) the limited applicability of a program to different MS platforms; (ii) practical factors such as file compatibility and data visualization; and (iii) the variations in the sample preparation protocols are critical aspects that drive the choice of a data analysis program [115]. Fig. 3 shows the open-source packages that are available for the different quantification methods.

#### 2.6.2 OpenMS

OpenMS includes several software packages to perform quantitative analysis for a particular technique, such as the *SILACAnalyser* [118], and *iTRAQAnalyser*, using the mzML data standard as a common input to all modules. In addition, the OpenMS team made improvements to the existing label-free quantification methods and algorithms, for the adjustment of the time scales and for the intelligent merging of related measurements of peptide and protein abundances.

#### 2.6.3 TPP

The TPP also provides different tools such as ASAPRatio [119] (for ICPL, ICAT, and SILAC techniques), SuperHirn [55] and SpecArray [120] for label-free methods, Libra [121], designed for iTRAQ approaches, and XPRESS [122] used for N^15^, ICPL, ICAT, and SILAC. The TPP package contains solutions and tools for most of the quantitation methods. In contrast with other TPP components, all the quantitation related libraries are written in C/C++ and have cross-platform support, which is important for their potential integration with other tools.

#### 2.6.4 Compomics

Rover [57,123] is a Java tool that facilitates the validation of regulated proteins found in MS-driven quantitative proteomics studies. The Mascot Distiller Quantitation toolbox creates by default a .rov file for each LC–MS/MS run analyzed, but only one .rov file can be opened and analyzed by MASCOT Distiller at a time, making it difficult to obtain a general view on protein quantification. Also, MaxQuant [124] creates text files as output that can open in Microsoft Excel, but analysis of results is generally difficult since no protein-specific visualization can be created. Rover accepts quantitative data from different sources such as Mascot Distiller and MaxQuant. In an intuitive environment, Rover visualizes these data such that the user can select and validate algorithm-suggested regulated proteins in the frame of the whole experiment and in the context of the protein inference problem.

#### 2.6.5 ProteoWizard and Skyline

Skyline [62,63] is a C# client tool and open-source framework for targeted proteomics and label-free quantitative methods. The framework uses the ProteoWizard libraries to import native output files from Agilent, Applied Biosystems, Thermo Fisher Scientific and Waters triple quadrupole instruments. The Skyline repository (http://proteowizard.svn.sourceforge.net/viewvc/proteowizard/trunk/pwiz/pwiz_tools/Skyline/) contains well-document examples about how to use the library. Another important feature of the tool is the vast community behind the platform, supported by the number of publications and the rich array of graphs available for inspecting data integrity.

#### 2.6.6 Other packages and open-source frameworks

*MSQuant* [125] (http://msquant.sourceforge.net/) is a *Microsoft* .NET software framework designed for quantification studies. It supports relative protein quantification based on precursor ion intensities, including element labels (N^15^), residue labels (SILAC and ICAT), termini labels (O^18^), functional group labels (mTRAQ), and label-free intensity approaches. Different proprietary file formats are supported, such as .RAW, .DAT, and .WIFF from Thermo, Waters, and Applied Biosystems, respectively. The library and tool allow the linking of Mascot result files with the corresponding raw data files. It also enables the user to specify the quantification mode used, or to set various filters for the parsing of the Mascot files, among many others parameters.

*MFPaQ* [126] is a Perl package dedicated to parse, validate, and quantify proteomics data coming from Mascot results. It supports data quantification using isotopic labeling methods (SILAC/ICAT) or label free approaches (spectral counting, MS signal comparison). The library provides the methods and functions to retrieve Mascot protein lists, sort them according to different Mascot parameters (such as the score and the rank order of the identified peptides), and to validate the results.

*X-Tracker* (http://www.x-tracker.info/) and *ProteoSuite* [127] (http://www.proteosuite.org/) are Java frameworks for the analysis of quantitative proteomics data. *X-Tracker* is able to support quantitation data coming from many different approaches, both at the MS or MS/MS level and its analysis workflow can be divided in four main steps: (i) loading of raw data and protein identifications; (ii) peak selection; (iii) computation of quantities; and (iv) reporting of the results. The software is distributed together with some pre-implemented modules to perform quantification using approaches like metabolic labeling, iTRAQ and label free techniques. *X-Tracker* is different from other platforms in the sense that it provides a plug-in based framework to support and extend some of the most common quantification methods (iTRAQ, TMT, N^15^ and emPAI [128]). The recently developed *ProteoSuite* tool is based on the plug-in architecture of *X-Tracker* and most of the features of the library come from *X-Tracker* itself. One of the key advantages of this tool is that can take as input files the standards mzML and mzIdentML. *IsobariQ* [129] is a software that employs the statistical software package R and variance stabilizing normalization (VSN) algorithms for relative quantification, which can be either based on the relative intensities of reporter ions in the low mass region (iTRAQ and TMT) or on the relative intensities of quantification signatures throughout the spectrum due to isobaric peptide termini labeling (IPTL).

### 2.7 Data storage and transfer to public data repositories

Although storing a few files on a file system is no longer a challenge for a small laboratory, with the increasing size of the data generated in each average experiment, it is crucial to organize and annotate data within local laboratory information management systems (LIMS), and/or in a public data repository. This can potentially solve four different problems: (i) files are poorly annotated experimentally; (ii) files are “organically” distributed across laboratory file systems in an *ad hoc* manner; (iii) files formats become obsolete; and (iv) searching the data and comparing results across separate experiments is very inefficient [21,130].

The common functionalities and use cases covered by a LIMS can be divided in: (i) how the framework acquires, presents, stores, and analyzes the data; (ii) they can have a one or two-way communication with a variety of other software components or instruments to receive the data; and (iii) they can have varying levels of privilege and access, which helps to prevent accidental modification or data loss, but external connections can also be enabled [131].

Once the experimental results are processed and can support the results described in a manuscript, it is considered to be a good practice to submit the data to a proteomics data repository [132]. This is increasingly recommended by several journals in the field like *Proteomics* or *Molecular and Cellular Proteomics* (MCP), among others. The main publicly available databases for MS proteomics data are the Global Proteome Machine Database (GPMDB) [133], PeptideAtlas [134], the PRIDE database [135] and Tranche (http://www.tranche.proteomecommons.org). PRIDE is a centralized, standard compliant, public data repository. It has been developed to provide the proteomics community with a public repository for protein and peptide identifications (also quantification is now supported), together with the mass spectra and the available metadata. It is important to highlight that data in PRIDE is not reprocessed in any way, while PeptideAtlas and GPMDB reprocess the data using the very popular pipelines TPP and X!Tandem, respectively.

PRIDE and PeptideAtlas are leading the ProteomeXchange consortium (http://www.proteomexchange.org) [136]. They are implementing a system to enable the automated and standardized submission and dissemination of MS-based proteomics data between the main existing MS proteomics repositories. PRIDE acts as the initial submission point for MS/MS data in the first implementation of the data workflow [137], while PeptideAtlas/PASSEL (PeptideAtlas SRM Experiment Library)[138] has an equivalent role for SRM data.

#### 2.7.1 Compomics

*ms_lims* [36] is a LIMS part of the compomics framework. It facilitates the import of mass spectra acquired from different mass spectrometers in MGF format and then stored in a relational database. It supports the parsing and storage of the results obtained from Mascot and it is completely integrated with the Mascot Daemon software, and also provides access to a Mascot server. The package (http://ms-lims.googlecode.com/) implements different filters and processing steps for peptide/protein identifications, and supports SILAC and iTRAQ approaches. *ms-lims* is currently undergoing a redevelopment process, and will soon be released with a new name: *colims* (http://colims.googlecode.com). The new *colims* application will result in a fully self-contained, freely available system for end-to-end MS based proteomics identification pipelines.

#### 2.7.2 PRIDE toolsuite

The PRIDE core Java API (http://ebi-pride.googlecode.com) can also be used as a basic LIMS using the *pride-core* and the *pride-web* source code libraries. In addition, using the PRIDE Converter 2 and PRIDE Inspector tools, the researcher can convert different files formats and do a basic analysis of the data locally.

The PRIDE Inspector tool can be used by the researchers to check the data before it is submitted to PRIDE. At present it supports mzML and PRIDE XML, but work to support mzIdentML is in progress. It contains different views on the data: (i) ‘Experiment overview’ includes uniform experimental metadata; (ii) ‘Protein view’ shows the information about the identified proteins and contains a powerful sequence viewer; (iii) ‘Peptide View’ shows the peptide identified highlighting the PTMs. In the Protein and Peptide views it is possible to vizualize MS/MS fragment ion annotations from each spectrum responsible of the identification; (iv) in the ‘Spectrum and Chromatogram’ view also unidentified spectra and chromatograms can be browsed (chromatograms are only present in mzML); (v) ‘Quantification view’ allows the visualization of quantification values for both protein and peptides. It is also possible to generate histograms where the expression values of up to ten proteins can be compared; and (vi) the ‘Summary charts view’ provides a collection of charts for assessing the overall properties of the data set, such as number of tryptic peptides, overall delta mass, number of missed cleavages sites, etc. Fig. 2B shows how the PRIDE Inspector tool can be used in combination with PRIDE Converter 2, before the submission to PRIDE is performed.

#### 2.7.3 Other packages and open-source frameworks

The *Proteios Software Environment* (ProSE) [139] is a web-based local data management system. ProSE has support for data coming from several quantitative proteomics workflows (TMT, iTRAQ), and integrates results from several search engines (Mascot, X!Tandem, OMSSA). The MS data is stored in the mzML and mzData formats, and can be exported to the PRIDE XML format. Additionally, it also provides a programming interface to enable local extensions, as well as database access using web services.

Finally, *MASPECTRAS* [50] is a web-based framework for the management and analysis of LC–MS data, which supports annotation standards like MIAPE (Minimum Information About a Proteomics Experiment). Some of the functionality included is: (i) importing and parsing of the results from the search engines Sequest, Mascot, Spectrum Mill, X!Tandem, and OMSSA; (ii) peptide validation using a linear discriminant score based on the database search scores; (iii) clustering of proteins based on Markov Clustering and multiple alignments; and (iv) quantification using the Automated Statistical Analysis of Protein Abundance Ratios algorithm (ASAPRatio).

### 2.8 Targeted proteomics: SRM.

Targeted proteomics approaches such as SRM constitute an attractive method to monitor a given set of proteins over various experimental conditions [140]. SRM, originally used for small-molecule MS, it is becoming the reference method for protein quantification in complex biological samples. Unlike LC–MS/MS, which requires computationally intensive bioinformatics post-analysis, targeted proteomics approaches require pre-acquisition bioinformatics analysis to determine: (i) the proteotypic peptides (peptides that have good ionization properties and are often detected in MS experiments), and (ii) optimal transitions (characteristic precursor and fragment ion combinations for a given peptide) to uniquely identify and to accurately quantify the proteins of interest. Extensive sets of bioinformatics tools, both web-based and stand-alone, have been developed to assist researchers to determine optimal peptides and transition sets. The proteotypic peptides and transitions are often selected based on the preferred precursor charge state, peptide sequence and molecular weight, hydrophobicity, fragmentation pattern at a given collision energy, and instrumentation used. In the next subsections we are going to give a brief overview of some of the existing tools. We recommend the following review focused on SRM computational resources [141], for getting more information.

#### 2.8.1 OpenMS

OpenMS contains a set of classes and components suited for SRM approaches. It can perform an optimal selection of transitions for a given set of proteins based on their sequence information alone or in conjunction with the already existing databases containing experimentally validated transitions. The method enables a rapid and fully-automated initial development of assays. The “PTModel” application is used to train a model for the prediction of proteotypic peptides. The input consists of two files: one file contains the positive examples (the peptides which are proteotypic) and the other contains the negative examples (the non-proteotypic peptides) [142]. The function is based on a support vector machine approach. “PTModel” will then perform a cross-validation to find the best combination of parameters, and then the resulting model is stored.

“PrecursorIonSelector” is a tool for precursor ion selection based on MS/MS identification results. The application uses the “FeatureFinder” module to identify “features” in a LC/MS map, where a feature is a peptide in a MS sample that reveals a characteristic isotope distribution. Given the map of features of the LC–MS run and the identification results, “PrecursorIonSelector” determines the next precursors [143].

#### 2.8.2 TPP

To compute accurate error rates, mProphet [56], a semi-supervised learning algorithm, is used for the identification of optimal target peptides. mProphet uses the “decoy transition concept” to maximize the separation of target and decoys peptides, thereby improving the confidence of the identifications.

mQuest [56] and ATAQS (Automated and Targeted Analysis with Quantitative SRM) [144] generate parameters for transition properties (e.g. retention time deviation, dot product of transition intensity between the light and heavy forms of the peptides, etc.) as a tool used before mProphet (Supplementary Information). As a unique feature to ATAQS, it provides an interface useful not only to select optimum transitions of given peptides, but also to select biologically relevant proteins using PIPE2 [145].

AuDIT [146] can automatically detect imprecise transitions for each peptide using the *t*-test and coefficient of variation between endogenous analytes and internal standard peptide transitions, if applicable. Both mProphet and AuDIT are automated modules that can be used to generate probability estimates for observed peptides and transition level accuracy. Another tool, SRMStat [147] employs user-filtered transitions and takes the transition quantification values to infer protein-level abundance changes by comparing the protein quantification level among classes of samples.

Finally, MaRiMba [148] is a framework to automate the creation of explicitly defined SRM transition lists required for triple quadrupole mass spectrometers. MaRiMba creates transition lists from spectral libraries, restricts the output to specified proteins or peptides, and filters the information based on precursor peptide and product ion properties. This open-source application is operated through a GUI incorporated into the TPP.

#### 2.8.3 Compomics

Sigpep (http://compomics-sigpep.googlecode.com) [149] provides transition redundancy analysis while calculating unique peptide signatures. The open-source software package retrieves all protein sequences from Ensembl and subsequently performs an *in silico* digestion using a protease of choice, allowing up to one missed cleavage. Then, all peptides are ordered by mass range and sequence uniqueness in order to select detectable proteotypic peptides for each protein of interest. Based on user-specified target proteins or peptides, the library will subsequently construct the expected transition background by *in silico* fragmentation of all isobaric peptides from the selected Ensembl database. The Sigpep application will then analyze and return a set of transitions that provide a unique signature against the expected background for each target peptide. Sigpep can be accessed using a web application.

#### 2.8.4 ProteoWizard and Skyline

Skyline is an application originally designed for the creation of methods for targeted proteomics. The Skyline user interface simplifies the development of MS methods and the analysis of data of SRM experiments. It supports the export of transition lists and imports the native output files from Agilent, Applied Biosystems, Thermo and Waters triple quadrupole instruments, seamlessly connecting the mass spectrometer output back to the experimental design document using the ProteoWizard package. The fast and compact Skyline file format is easy to share. As a key feature, multiple graphs are generated for inspecting data integrity during the data acquisition process, helping instrument operators to identify problems early.

Skyline provides several ways of building and editing SRM methods and models. Users can copy the protein sequences or lists of peptides, precursors and product ion transitions either into a dialog, or directly into the document. Additionally, transition lists and results, for private and published experiments on MRMer (see next section) [150] are easily recreated in Skyline.

#### 2.8.5 Other packages and open-source frameworks

MRMer [150] allows users to accept and/or reject transitions by manual selection and automated analysis of transitions. Additionally, it allows users to interactively select the start and stop retention times that can be used for quantification for a given transition, and to manually select/unselect verified transitions for a given peptide ion. MRMaid [151] offers an alternative for the design of SRM transitions using a combination of knowledge of the properties of optimal SRM transitions taken from expert practitioners, data stored in PRIDE [152] and literature with MS/MS evidence. The tool also predicts retention time values using a published model, since transition candidates are ranked based on a novel transition scoring system. Users may then filter the results by selecting optional stringency criteria, such as taking into account frequently modified residues, constraining the length of peptides, or omitting missed cleavages.

## 3 Conclusions

Open-source frameworks and libraries play an important role in the development and growth of the new MS-based proteomics tools. As a matter of fact, they can greatly simplify the implementation of the basic features needed in most tools and allow the developers to focus on the novel aspects, rather than on the basic functions, which can contribute substantially to achieve a faster development. Basic and complex functionalities are both supported, such as protein sequence digestion, sequence feature predictions, file format readers and converters, spectrum preprocessing and peptide/protein post-processing, among others.

OpenMS [33], Trans Proteomic Pipeline (TPP), Compomics [35,38,39,89–91], ProteoWizard [42], the Java Proteomic Library [43,44], the PRIDE toolsuite [40,41,64,86–88] and msInspect [49] contain some of the most extensively and complete libraries used by the proteomics community. Most of them are written in Java, C++, Perl, and Python. Finally, it is worth mentioning that msCompare [153] is a good example of the use and integration of different MS software packages such as OpenMS, SuperHim and mzMine. Further improvements in the integration, development and documentation must be considered by the computational proteomics community in order to facilitate the reuse of the current software libraries available.

The open-source libraries and frameworks described in this review have been fundamental in building new bioinformatics tools. In fact, there has been a big progress in the development of new libraries, allowing them to be folded into other applications and pipelines as reusable building blocks, and answer different research questions. One of the reasons behind is that the development of open source software offers the potential for a more flexible technology and potentially, quicker innovation. One of the known downsides is the lack of a thorough documentation in some cases, which may cause that the software cannot be easily reused. Since bioinformatics has become such a fundamental part of proteomics research, future work will continue to expand these libraries and frameworks to provide more powerful and robust analysis tools.

## Figures and Tables

**Table 1:** Different libraries for *in silico* analysis of proteins. Isoelectric point (*pI), retention time (RT),* Sequence Digestion (*SD*), Decoy database generation (*DDG*), consider post-translational modifications (*PTM*), molecular formula prediction (*MFP*), FASTA Sequence Databases Reader (FD).

Library	Language	Version	Property prediction	Custom features	Supported formats	URL	Integration	Reference
BioJava	Java	Legacy 1.8.2 (2012)	*pI*, Mass, AAIndex	SD	FD	http://www.biojava.org	Maven	[154]
compomics-utilities	Java	3.6.12 (2012)	RT, GRAVY index, isotopic distribution	SD, PTM, Sequence pattern filtering, Decoy DDG	FD, Mascot dat, X!Tandem XML, OMSSA output, Proteome Discoverer/ msf files	http://compomics-utilities.googlecode.com	Maven	[35]
InsilicoSpectro	Perl	1.3.24 (2008)	RT, *pI*, mass, hydrophobicity	SD, PTM	FD, Mascot XML output	http://search.cpan.org/~alexmass/InSilicoSpectro	CPAN	[45]
Java Proteomic Library (JPL)	Java	1.0 (2012)	*pI*, mass, hydrophobicity, GRAVY index, charge and specific pH	SD, PTM, MFP	FD	http://javaprotlib.sourceforge.net	–	
mspire	Ruby	0.8.2 (2012)	Mass, isotopic distribution	SD, MFP	FD	http://github.com/princelab/mspire	–	[155]
multiplierz	Python	(2011)	Mass	SD	FD	http://blais.dfci.harvard.edu/index.php?id=63	–	[46]
OpenMS	C++	1.9 (2012)	Mass, RT	SD, PTM, DDG	FD, Mascot XML output	http://open-ms.sourceforge.net	–	[33]
pyteomics	Python	1.2.5 (2012)	*pI*, Mass, charge, isotopic distribution, RT	SD	FD	http://pypi.python.org/pypi/pyteomics	PyPI	
TPP (Trans Proteomic Pipeline)	C++, Java	4.6 (2012)	*pI*, mass	SD, PTM, Proteotypic Peptide Prediction, DDG	FD	http://sourceforge.net/projects/sashimi/files/Trans-Proteomic%20Pipeline%20%28TPP%29	–	[156]

**Table 2:** Software libraries to read (r) and write (w) MS-based information from different file formats.

Library	Language	File formats									URL	Integration	Reference
		mzML	mzXML	mzData	Peak list files	Search engine output files	mzIdentML	mzTab	FASTA	PRIDE XML			
compomics-utilities	Java	–	–	–	r/w (mgf)	r (OMSSA, Mascot, X!Tandem)	–	–	r/w	r/w	http://compomics-utilities.googlecode.com	Maven	[35,39,89,90]
jmzIdentML	Java	–	–	–	–	–	r/w	–	–	–	http://jmzidentml.googlecode.com	Maven	[87]
jmzML	Java	r/w	–	–	–	–	–	–	–	–	http://jmzml.googlecode.com	Maven	[86]
jmzReader	Java	r	r	r	r (mgf, pkl, ms2, dta)	–	–	–	–	–	http://jmzreader.googlecode.com	Maven	[88]
jmzTab	Java	–	–	–	–	–	–	r/w	–	–	https://mztab.googlecode.com	Maven	
JRAP	Java	–	r/w	–	–	–	–	–	–	–	http://tools.proteomecenter.org/wiki/index.php?title=Software:JRAP	–	
MGFp	C++	–	–	–	–	r Mascot	–	–	–	–	http://sourceforge.net/projects/mgfp	–	[94]
OpenMS	C++	r/w	r/w	r/w	–	r (Mascot, Sequest, OMSSA, X!Tandem)	–	–	r	–	http://open-ms.sourceforge.net	–	[33]
PRIDE Converter 2	Java	r	r	r	r (mgf, pkl, ms2, dta)	r (Mascot, X!Tandem, OMSSA, SpectraST, CRUX, MSGF, Proteome Discoverer)	r	–	r	r/w	http://pride-converter-2.googlecode.com	Maven	[64]
ProteoWizard	C++	r/w	r/w	–	r/w (mgf, ms2)	–	r/w	–	–	–	http://proteowizard.sourceforge.net	–	[42]
pymzML	Python	r/w	–	–	–	–	–	–	–	–	http://pymzml.github.com	pypi	

**Table 3:** Different software packages to pre-processing the MS proteomics and metabolomics data.

Library	Language	File formats	Processing Methods					URL	Reference
			Spectrum normalization	Spectrum clustering	Deconvolution	Spectrum alignment	Spectrum quality assessment		
maltcms	Java	mzML, mzXML, mzData	X		X			http://maltcms.sourceforge.net/home/index.html	[98]
mMass	Python	mzML, mzXML, mzData, MGF,	X		X			http://www.mmass.org	[47]
msInspect	Java	mzXML			X		X	http://proteomics.fhcrc.org/CPL/msinspect/index.html	[49]
mzMine2	Java	mzML, mzXML. mzData	X					http://mzmine.sourceforge.net	[48]
OpenMS	C++	mzML, mzXML, mzData	X	X		X		http://open-ms.sourceforge.net	[33]
