# Supplementary material for: Open source libraries and frameworks for mass spectrometry based proteomics: A developer's perspective
Source: Biochim Biophys Acta. 2014 Jan;1844(1):63–76. doi: 10.1016/j.bbapap.2013.02.032 (PMC3898926; doi:10.1016/j.bbapap.2013.02.032)
Supplement: Supplementary file 1 — Supplementary material. [file mmc1.docx]

**Code example 1:** Protein digestion using the Java Proteomic Library.

import static org.junit.Assert.assertEquals;

import java.text.ParseException;

import java.util.ArrayList;

import java.util.Arrays;

import java.util.HashSet;

import java.util.Iterator;

import java.util.List;

import java.util.Set;

import org.apache.commons.collections15.Transformer;

import org.expasy.jpl.commons.base.cond.Condition;

import org.expasy.jpl.commons.base.cond.ConditionImpl;

import org.expasy.jpl.commons.base.cond.operator.impl.OperatorLowerThan;

import org.expasy.jpl.core.mol.chem.ChemicalFacade;

import org.expasy.jpl.core.mol.modif.ModificationFactory;

import org.expasy.jpl.core.mol.polymer.pept.Peptide;

import org.expasy.jpl.core.mol.polymer.pept.matcher.AAMotifMatcher;

import org.expasy.jpl.core.mol.polymer.pept.rule.EditionRule;

import org.expasy.jpl.core.mol.polymer.pept.rule.PeptideEditorFactory;

import org.expasy.jpl.core.mol.polymer.pept.rule.EditionRule.EditionAction;

import org.expasy.jpl.core.mol.polymer.pept.term.CTerminus;

import org.expasy.jpl.core.mol.polymer.pept.term.NTerminus;

public class JPLDigesterTest{

private Peptide sequence;

private Peptidase trypsin;

private Digester digester;

public void digester() throws Exception {

sequence = new Peptide.Builder("RESALYTNIKALASKR").build();

trypsin = Peptidase.getInstance("Trypsin");

digester = Digester.newInstance(trypsin);

digester.setNumberOfMissedCleavage(2);

digester.digest(sequence);

Iterator<DigestedPeptide> iter = digester.getDigests().iterator();

while (iter.hasNext()) {

DigestedPeptide digest = iter.next();

}

}

} //end of main

**Code example 2:** Conversion of a peak list file to an mzML file using ProteoWizard.

//

#include "pwiz/data/msdata/MSDataFile.hpp"

#include "pwiz/utility/misc/SHA1Calculator.hpp"

#include "pwiz/utility/misc/Filesystem.hpp"

#include "pwiz/utility/misc/Std.hpp"

using namespace pwiz::cv;

using namespace pwiz::data;

using namespace pwiz::util;

using namespace pwiz::msdata;

namespace bfs = boost::filesystem;

void flush(SpectrumListSimple& sl, const string& nativeID, const string& msLevel,const vector<MZIntensityPair>& pairs){

// fill in a new Spectrum and append it to the SpectrumList

SpectrumPtr spectrum(new Spectrum);

spectrum->index = sl.size();

spectrum->id = "scan=" + nativeID;

spectrum->set(MS_ms_level, msLevel);

spectrum->setMZIntensityPairs(pairs, MS_number_of_counts);

sl.spectra.push_back(spectrum);

}

void peak2mzml(const char* filenameIn, const char* filenameOut){

// open input file

ifstream is(filenameIn);

if (!is)

throw runtime_error(("[txt2mzml] Unable to open file " + string(filenameIn)).c_str());

MSData msd;

SpectrumListSimplePtr sl(new SpectrumListSimple);

msd.run.spectrumListPtr = sl;

SourceFilePtr sourceFile(new SourceFile);

bfs::path p(filenameIn);

sourceFile->id = "text_data";

sourceFile->name = BFS_STRING(p.leaf());

sourceFile->location = string("file://") + BFS_COMPLETE(p.branch_path()).string();

string sha1 = SHA1Calculator::hashFile(filenameIn);

sourceFile->cvParams.push_back(CVParam(MS_SHA_1, sha1));

msd.fileDescription.sourceFilePtrs.push_back(sourceFile);

msd.run.id = "run";

string nativeID;

string msLevel;

vector<MZIntensityPair> pairs;

while (is){

string buffer;

getline(is, buffer);

if (!is){

flush(*sl, nativeID, msLevel, pairs);

break;

}

istringstream iss(buffer);

vector<string> tokens;

copy(istream_iterator<string>(iss), istream_iterator<string>(), back_inserter(tokens));

if (tokens.empty() || tokens[0]=="#") continue;

if (tokens.size() != 4 || tokens[1].size()<3 || tokens[1].substr(0,2)!="ms")

throw runtime_error((buffer + "\n[txt2mzml] Bad format.").c_str());

if (tokens[0] != nativeID){

if (!nativeID.empty()) flush(*sl, nativeID, msLevel, pairs);

nativeID = tokens[0];

msLevel = tokens[1].substr(2);

pairs.clear();

}

pairs.push_back(MZIntensityPair(lexical_cast<double>(tokens[2]),lexical_cast<double>(tokens[3])));

}

MSDataFile::write(msd, filenameOut);

}

} //end of function

**Code example 3:** Reduction of noise and baseline background using OpenMS.

[Int](file:///C:\Program%20Files\OpenMS-1.8\doc\classInt.html) [main](file:///C:\Program%20Files\OpenMS-1.8\doc\AdditiveSeries_8C.html#217dbf8b442f20279ea00b898af96f52)()

{

PeakMap exp;

MzMLFile mzdata_file;

mzdata_file.load("data/Tutorial_GaussFilter.mzML",exp);

GaussFilter g;

Param param;

param.setValue("gaussian_width",1.0);

g.setParameters(param);

g.filterExperiment(exp);

return 0;

} //end of main

**Code example 4:** Function to check the mass delta in a Mascot .dat output file using Peptizer.

package com.compomics.peptizer.util.agents;

import com.compomics.mascotdatfile.util.interfaces.MascotDatfileInf;

import com.compomics.mascotdatfile.util.mascot.MascotDatfile;

import com.compomics.mascotdatfile.util.mascot.PeptideHit;

import com.compomics.mascotdatfile.util.mascot.Query;

import com.compomics.mascotdatfile.util.mascot.enumeration.MascotDatfileType;

import com.compomics.mascotdatfile.util.mascot.factory.MascotDatfileFactory;

import com.compomics.peptizer.interfaces.Agent;

import com.compomics.peptizer.util.AgentFactory;

import com.compomics.peptizer.util.AgentReport;

import com.compomics.peptizer.util.PeptideIdentification;

import com.compomics.peptizer.util.datatools.implementations.mascot.MascotPeptideHit;

import com.compomics.peptizer.util.datatools.implementations.mascot.MascotSpectrum;

import com.compomics.peptizer.util.enumerator.AgentVote;

import com.compomics.peptizer.util.enumerator.SearchEngineEnum;

import java.io.File;

import java.util.Vector;

public void testDeltaMassPPM() {

Agent lAgent = AgentFactory.getInstance().getAgent("com.compomics.peptizer.util.agents.DeltaMassPPMAgent");

String datFile = getFullFilePath("F015264_small.dat");

if (File.separatorChar == '\\') datFile = datFile.replace("%20", " ");

MascotDatfileInf lMascotDatfile = MascotDatfileFactory.create(datFile, MascotDatfileType.INDEX);

int lQueryNumber = 1;

Query lQuery = (Query) lMascotDatfile.getQuery(lQueryNumber);

Vector lPeptideHits = lMascotDatfile.getQueryToPeptideMap().getAllPeptideHits(lQueryNumber);

Vector lPeptizerPeptideHits = new Vector(lPeptideHits.size());

for (int i = 0; i < lPeptideHits.size(); i++)

lPeptizerPeptideHits.add(new MascotPeptideHit((PeptideHit) lPeptideHits.get(i), i + 1));

MascotSpectrum mascotSpectrum = new MascotSpectrum(lMascotDatfile.getQuery(lQueryNumber));

PeptideIdentification lPeptideIdentification = new PeptideIdentification(mascotSpectrum, lPeptizerPeptideHits, SearchEngineEnum.Mascot);

lAgent.setProperty(DeltaMassPPMAgent.TOLERANCE, "100");

AgentVote[] lResult = lAgent.inspectIfPossible(lPeptideIdentification);

Assert.assertEquals(1, lResult[0].score);

Assert.assertTrue(lResult.length == 3);

Assert.assertEquals(-136.58025914779105, lPeptideIdentification.getAgentReport(1, lAgent.getUniqueID()).getReport(AgentReport.RK_ARFF));

Assert.assertEquals("-136.5803", lPeptideIdentification.getAgentReport(1, lAgent.getUniqueID()).getReport(AgentReport.RK_TABLEDATA).toString());

Assert.assertEquals(AgentVote.POSITIVE_FOR_SELECTION, lPeptideIdentification.getAgentReport(1, lAgent.getUniqueID()).getReport(AgentReport.RK_RESULT));

Assert.assertEquals(322.720322017764, lPeptideIdentification.getAgentReport(3, lAgent.getUniqueID()).getReport(AgentReport.RK_ARFF));

Assert.assertEquals("322.7203", lPeptideIdentification.getAgentReport(3, lAgent.getUniqueID()).getReport(AgentReport.RK_TABLEDATA).toString());

Assert.assertEquals(AgentVote.POSITIVE_FOR_SELECTION, lPeptideIdentification.getAgentReport(3, lAgent.getUniqueID()).getReport(AgentReport.RK_RESULT));

}

//end of main

**Code example 5:** Transition Generator using ATQS.

import java.io.IOException;

import java.util.List;

import org.systemsbiology.lib.commonobj.*;

public class TransitionGenerator {

public static final String INPUT_TEST_FILE= "/users/skwok/Desktop/inputTransition.csv";

public static final String OUTPUT_TEST_FILE= "/users/skwok/Desktop/outputTransition.csv";

public static void main(String args[]){

//Factory class get Transition parser which parse full format (csv)

ITransitionParser parser = TransitionParserFactory.getInstance(TransitionParserFactory.FULL_FORMAT);

//Factory class get decoy algorithm

IDecoyAlgorithm algor= DecoyAlgorithmFactory.getInstance(DecoyAlgorithmFactory.SIMPLE_ALGORITHM);

DecoyAlgorithmBean bean = DecoyAlgorithmBean.getDefaultInstance();

DecoyTransitionGenerator gen = new DecoyTransitionGenerator(INPUT_TEST_FILE, algor, parser);

try{

String name = parser.getTransitionParserName();

parser.parse(INPUT_TEST_FILE);

List<Transition> t = parser.getTransitions();

System.out.println("print each transition");

for(Transition tt : t) System.out.println(tt);

System.out.println("\nthe total transition: " + t.size());

System.out.println("print each decoy transition");

List<Transition> decoy_t = gen.makeDecoyTransition();

for(Transition tt : decoy_t) System.out.println(tt);

System.out.println("\nthe total transition: " + decoy_t.size());

System.out.println("write decoy transition to a file");

ExportType.FULL.write(decoy_t, OUTPUT_TEST_FILE);

}catch(IOException e){

e.printStackTrace();

}

}

}

//end of main
